# Vascular assessment techniques of podiatrists in Australia and New Zealand: a web-based survey

**DOI:** 10.1186/s13047-015-0130-5

**Published:** 2015-12-09

**Authors:** Peta Ellen Tehan, Vivienne Helaine Chuter

**Affiliations:** School of Health Sciences, Faculty of Health, University of Newcastle, Ourimbah, 2258 NSW Australia

**Keywords:** Non-invasive vascular assessment, Podiatrist, Survey, Clinical practice

## Abstract

**Background:**

Podiatrists play a central role in conducting non-invasive vascular assessment in the lower extremity. This involves screening for signs and symptoms of peripheral arterial disease (PAD) and ongoing monitoring of the condition. Podiatric vascular assessment practices in Australia and New Zealand are currently unclear. Determining the clinical habits of Podiatrists is essential in identifying if there is a need for further education or support in performing accurate vascular assessments.

**Methods:**

A web-based, secure, anonymous questionnaire was conducted of registered Podiatrists in Australia and New Zealand between 1 April and 31 July 2013. The questions examined clinician’s regular practices in vascular assessment, clinical indicators to perform and barriers in completing vascular assessment. Nominal logistic regression was performed to further examine years of experience and practice setting on clinical indicators to perform vascular assessment and types of assessment performed.

**Results:**

Four hundred forty-seven podiatrists participated in the survey. Clinical indicators for vascular assessment, along with barriers and available equipment were examined and the results varied depending on the podiatrists’ geographical location, practice setting, and experience. Palpation of pedal pulses was the most frequently reported assessment (97 %) along with Doppler assessment (74 %). Pressure measurement was the least frequently reported vascular assessment method, with only 34 % undertaking ankle-brachial indices and 19 % completing toe-brachial indices. Public podiatrists reported more varied and complete vascular assessment compared to those in private practice. Lack of time was identified as the most frequently reported barrier (66 %) in performing vascular assessment, followed by lack of equipment (28 %). In New Zealand podiatrists, lack of equipment was much more of an issue than in Australian podiatrists.

**Conclusion:**

Large variations exist in vascular assessment methods amongst Australian and New Zealand podiatrists. Some assessments being undertaken are potentially inadequate for accurate screening for PAD. There is a need for continuing education in vascular assessment to address the deficiencies in technique reported by some Podiatrists. A podiatry-relevant summary of broad international guidelines for PAD screening may be of use to improve utilisation and accuracy of screening methods to improve patient management.

**Electronic supplementary material:**

The online version of this article (doi:10.1186/s13047-015-0130-5) contains supplementary material, which is available to authorized users.

## Background

Podiatrists play a central role in conducting non-invasive vascular assessment in the lower extremity. This involves screening for signs and symptoms of peripheral arterial disease (PAD) and ongoing monitoring of the condition following diagnosis [1]. Given that people with PAD are not only at higher risk of wounds and limb loss, but are at far greater risk of cardiovascular events and death [2], effective routine vascular assessment and subsequent accurate diagnosis of PAD is integral to improving clinical outcomes and to facilitate effective intervention and ongoing monitoring [3].

A number of tests are currently used for lower limb vascular assessment including pulse palpation, systolic toe pressures, toe-brachial index (TBI), ankle-brachial index (ABI) and Doppler examination. While generally these tests have been shown to have high reliability and diagnostic accuracy [4–12], there has been little investigation of the frequency of use and practicality of performing these assessments in clinical practice generally, with most evidence relating to the most widely recommended test, the ABI [13].

In general medical practice, time constraints and lack of financial reimbursement have been reported to contribute to reduced utility of the ABI for vascular screening [14] with general medical practitioners also reporting a lack of confidence in ability to perform the measurement [15]. Only 32% of general medical practitioners are reported to perform ABI on a regular basis most commonly prior to the application of compression bandaging and for determining the aetiology of chronic wounds [14]. Podiatrists also have reported time constraints and lack of financial reimbursement as barriers in performing ABI, with approximately half of practitioners reporting using ABI regularly [16]. However the clinical indicators used by clinicians to complete this assessment or conduct other forms of lower limb vascular assessment including the TBI and Doppler waveform assessment have not been investigated [15, 16].

The primary aim of this study was to determine current practices in performing lower limb vascular assessments of Podiatrists in Australia and New Zealand. The secondary aims of this study were to investigate factors influencing lower limb vascular assessment practices including levels of clinical experience and education, practice location and resources and to establish perceived barriers to performing lower limb vascular assessments Podiatry practice.

## Methods

This was a cross-sectional observational study performed using a web –based, secure anonymous self-administered survey reading lower limb vascular assessment techniques of Podiatrists from Australia and New Zealand that was conducted between 1 April and 31 July 2013.

Recruitment of participants was via their affiliated professional body—The Australian Podiatry Association or PodiatryNZ. Invitations to participate were sent via e-mail advertising in the weekly bulletin or a small advertisement in the paper based bulletin with a link to the survey. External clinical supervisors participating in the University of Newcastle external placement program were also invited to take part via email invitation containing a survey overview with a hyperlink to the survey. Inclusion criteria were Podiatrists registered and currently practicing in Australia and New Zealand. Ethical approval was obtained from the University of Newcastle Human Research Ethics Committee (Ethics approval: H-2012-0384). All participants provided informed consent prior to participation in this study.

The survey was delivered online via the online survey software Survey Monkey®. The questions examined clinician’s regular practices in vascular assessment, factors prompting performance of an assessment and availability of equipment (Additional file 1). The first seven questions elicited demographic and descriptive data from the participants. Questions eight to 15 related to clinicians vascular assessment habits and 16 and 17 related to provision of patient education. The majority of questions were closed with three open ended questions, which related to time spent in practice and topics covered in education provision. A mix of nominal polytomous, ordinal polytomous and dichotomous questions were used. Pilot testing of the survey was performed at a University of Newcastle continuing professional development event attended by a mix of 35 private and public sector podiatrists. Based on feedback from podiatrists some small amendments were made to some of the questioning methods from open ended to ordered polytomous and phrasing of the questions was slightly altered to allow for further clarity.

### Data analysis

The primary data analyses were descriptive statistics of the cohort including geographical practice location, years of experience, qualifications held and practice sector. Nominal logistic regression was performed and relative risk ratios calculated for possible factors affecting clinical indications to perform vascular assessment and the type of vascular testing that was performed. These clinical indicators included combinations of the type of referral received, clinical signs and symptoms of PAD and patient medical history. Vascular assessment performed included combinations of clinical observations, Doppler use and pressure measurements. The fit of the data to the final nominal logistic regression model was assessed using the Homser-Lemeshow test with a *p* value >0.05 indicating an adequate fit. All data analysis was conducted using Stata data analysis and statistical software version 13. Missing data were excluded case wise.

## Results

### Participant characteristics

Four hundred and forty seven podiatrists were recruited in total, however the number of responses varied slightly per question with some respondents not answering all questions, and some questions allowed for multiple answer options. Overall percentages are reported as the percentage of the total number of participants who answered an individual question and the total number of respondents for the question provided. Overall percentages are reported as the percentage of the total number of participants who answered an individual question. For comparison of sub-groups descriptive statistics are reported as the percentage of the number of respondents identified in that sub group e.g. practitioners in private practice. The total response rate represents approximately 10 % of all registered Podiatrists in Australia and New Zealand in 2013. Participant characteristics are included in Table 1.

**Table 1:** Survey participant characteristics

Participant characteristics	
Participants	447
Private practice	322 (73 %)
Public practice	115(26 %)
Research/education	10 (2 %)
Metropolitan area	265 (60 %)
Regional area	137 (31 %)
Rural area	57 (13 %)
Years of practice (Range)	0–42
Years of practice (Mean)	13
Diploma	80 (18 %)
Bachelor or equivalent	268 (61 %)
Post graduate qualification/Research Higher Degree	91 (21 %)

### Indicators to perform a vascular assessment

**Fig. 1:**
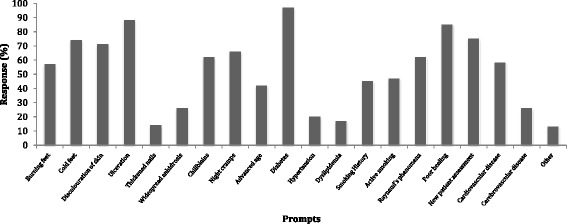
Clinical indicators for podiatrists to perform vascular assessment

A history of diabetes was the most frequently reported clinical indicator to complete a vascular assessment (82 %, *n* = 367/377) the least frequently reported was presence of thickened nails (14.6 %, *n* = 55/377) (Fig. 1). Several other cardiovascular risk factors for PAD including hypertension and dyslipidaemia were among the least frequently reported clinical indicators. The mean number of vascular assessments performed in the most recent day of practice was 2.35 and 10 min was the most frequently reported average time taken to complete vascular assessment (Table 2). The most commonly reported clinical indicators to perform a vascular assessment were grouped into the patient’s medical history, practitioner’s clinical observations and the type of referral i.e. Medicare EPC referral, general practitioner referral (Table 3).

**Table 2:** General vascular assessment information

General vascular assessment				
Mean number of vascular assessments performed in most recent day of clinical practice			2.35	
Vascular assessment within standard consultation *n* (%)			277 (73)	
Vascular assessment as separate consultation *n* (%)			47 (12)	
Charge additional fee for vascular assessment *n* (%)			34 (9)	
Do not charge additional fee for vascular assessment *n* (%)			280 (74)	
Time to complete assessment *n* (%)				
5 min	10 min	15 min	20 min	30 min
97 (25)	130 (34)	80 (21)	40 (12)	26 (7)

**Table 3:** Clinical indicators for vascular assessment

Clinical indicators	Medical history					Medical history and observations					Medical history, observations and referral type		Medical history and referral type				
	*N*	%	RRR	*P* value	95 % CI	*N*	*%*	RRR	*P* value	95 % CI	*N*	%	*N*	%	RRR	*P* value	95 % CI
Education level^a^																	
Diploma	6	8.45	0.93	0.789	0.55 to 1.569	13	18.31	0.78	0.251	0.51 to 1.189	47	66.2	5	7.04	1.40	0.44	06 to 3.282
Bachelor	30	11.95				33	13.15				150	59.76	38	15.14			
Postgrad/RHD	5	5.68				11	12.5				53	60.23	19	21.59			
Practice setting^b^																	
Private	30	10.38	**0.02**	**<0.0001**	0.003 to 0.153	52	17.99	**0.38**	**<0.0001**	0.22 to 0.652	162	56.06	45	15.57	**0.10**	**0.028**	0.01 to 0.782
Public	9	8.82				4	3.92				74	72.55	15	14.71			
Geographical location																	
Metro	21	8.57	2.05	0.292	0.54 to 7.773	40	16.33	0.96	0.945	0.27 to 3.430	149	60.82	35	14.29	2.38	0.345	0.39 to 14.435
Regional	16	12.21	0.71	0.609	0.2 to 2.592	15	11.45	0.36	0.11	0.11 to 1.258	81	61.83	19	14.5	1.35	0.731	0.24 to 7.640
Rural	4	7.69	1.15	0.831	0.31 to 4.304	4	7.69	0.94	0.927	0.27 to 3.249	33	63.46	11	21.15	2.77	0.244	0.5 to 15.394
Experience																	
Years (mean, SD)	12.01	8.96	**1.04**	**0.018**	1.01 to 1.073	14.82	11.14	**1.04**	**0.004**	1.01 to 1.066	12.14	10.04	13.60	9.73	**1.06**	**0.039**	1.00 to 1.117

*Values in bold are considered statistically significant, RRR = relative risk ratio

The reference group of the nominal logistic regression model used a combination of responses of Observations, Medical History and Referral Type

^a^Bachelor or equivalent degree was used as the reference category for education level

^b^Private practitioners were used as the reference category for work setting

Regression analysis showed the clinical indicators used as a basis for performing a vascular assessment were most strongly influenced by the years of clinical experience and practice setting (public of private) (Table 3). Public sector podiatrists were more likely to perform vascular assessment based on a combination of medical history, observations and the type of referral compared to private sector practitioners (*p* = <0.0001). Less experienced podiatrists were more likely to use a combination of multiple factors (referral type, medical history and observations) to prompt for vascular assessment (*p* = 0.018) compared to more experienced podiatrists who reported relying upon one or two clinical indicators alone, rather than a combination of all three clinical indicators. The Hosmer-Lemeshow test was identified as statistically non significant (*p* = 0.17) indicating the model was an adequate fit to the data.

### Vascular assessment methods

**Fig. 2:**
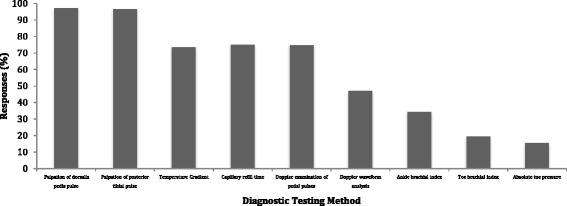
Diagnostic testing methods employed by podiatrists

Pedal pulse palpation (97 %, *n* = 366/377) and Doppler use (74 %, *n* = 281/377) were the most frequently reported vascular assessment tests by all respondents (Fig. 2). Use of vascular pressure measurement was substantially lower with 34.2 % (*n* = 129/377) of all respondents reporting regularly using ABIs and 19.4 % (*n* = 73/377) using TBIs. Public sector podiatrists reported a higher frequency of Doppler use (92 %, *n* = 101/110) than private-sector podiatrists (66 %, *n* = 197/300). There were also differences in frequency of use of pressure measurement between public and private sector podiatrists. Fifty three percent of public sector podiatrists reported regularly using an ABI (*n* = 58/110) and 35% regularly using a TBI (*n* = 39/110) whereas in the private sector, 25 % of podiatrists reported regularly using an ABI (*n* = 75/300) and only 12 % regularly used a TBI (*n* = 24/300). Nominal regression analysis revealed that setting (private or public sector) and years of experience were significant predictors of what testing methods were reported to be performed (Table 4). Private sector practitioners were less likely to use multiple assessments that included observations and Doppler (*p* = <0.0001) or observations and pressure measurement (*p* = 0.01), compared to public sector practitioners. More experienced podiatrists were also more likely to report relying on their clinical observations (*p* = 0.018) rather than undertaking clinical testing such as Doppler and pressure measurement to perform a lower limb vascular assessment.

**Table 4:** Types of testing utilised by podiatrists

Types of testing	Observations alone					Observations and doppler					Observations doppler and pressure		Observations and pressure				
	*N*	%	RRR	*P* value	95 % CI	*N*	%	RRR	*P* value	95 % CI	*N*	%	*N*	%	RRR	*P* value	95 % CI
Education level^a^																	
Diploma	19	26.76	0.93	0.789	0.55 to 1.569	32	45.07	0.78	0.251	0.51 to 1.189	17	23.94	3	4.23	1.40	0.44	06 to 3.282
Bachelor	43	17.2				92	36.8				107	42.8	8	3.2			
Postgrad/RHD	15	17.05				24	27.27				42	47.73	7	7.95			
Practice setting^b^																	
Private	70	24.31	**0.02**	**<0.0001**	0.003 to 0.153	115	39.93	**0.38**	**<0.0001**	0.22 to 0.652	89	30.9	14	4.86	**0.10**	**0.028**	0.01 to 0.782
Public	1	0.98				30	29.41				70	68.63	1	0.98			
Geographical location																	
Metro	53	21.72	2.05	0.292	0.54 to 7.773	98	40.16	0.96	0.945	0.27 to 3.430	82	33.61	11	4.51	2.38	0.345	0.39 to 14.435
Regional	20	15.27	0.71	0.609	0.2 to 2.592	34	25.95	0.36	0.11	0.11 to 1.258	71	54.2	6	4.58	1.35	0.731	0.24 to 7.640
Rural	8	15.38	1.15	0.831	0.31 to 4.304	20	38.46	0.94	0.927	0.27 to 3.249	21	40.38	3	5.77	2.77	0.244	0.5 to 15.394
Experience																	
Years (mean, SD)	14.4	8.3	**1.04**	**0.018**	1.01 to 1.073	14.5	11.4	**1.04**	**0.004**	1.01 to 1.066	10.1	9.0	15.5	10.1	**1.06**	**0.039**	1.00 to 1.117

*Values in bold are considered statistically significant, RRR = relative risk ratio

The reference group of the nominal logistic regression model used a combination of responses of Observations, Doppler and Pressure measurement

^a^Bachelor or equivalent degree was used as the reference category for education level

^b^Private practitioners were used as the reference category for work setting

### Barriers in performing vascular assessment

Time constraints were the most frequently nominated barrier to performing a vascular assessment for all respondents (62 %, *n* = 233/376), followed by general lack of equipment (28 %, *n* = 106/376). Lack of equipment was more frequently reported as a barrier in New Zealand podiatrists 43.8 % (*n* = 28/64) than their Australian counterparts (25 %, *n* = 78/312). No barriers to completing vascular assessment was reported by 22 % (*n* = 99/376) of the responding participants.

Private sector podiatrists reported time constraints were a barrier to performing vascular assessments (64 %, *n* = 190/293) more frequently than those in public practice (54 %, *n* = 58/108). Lack of equipment and uncertainty about technique were also more frequently reported in by podiatrists in private practice (equipment:32 %, *n* = 93/293, technique: 13 %, *n* = 38/293) than in public practice (equipment: 22 %, *n* = 24/108, technique: 3.7 %, *n* = 4/108).

Geographical location appeared to have an influence on barriers in performing vascular assessment. Although time constraints were the most commonly reported barrier in performing vascular assessment for all respondents (62 %, *n* = 233/376), this was highest amongst rural (77 %, *n* = 41/53), and regional podiatrists (62 %, *n* = 80/129) compared to those in metropolitan areas (58 %, *n* = 138/239). The majority of podiatrists unsure of assessment techniques were rurally located (17 %, *n* = 9/53), followed by those in metropolitan (10 %, *n* = 24/239) and regional (8 %, *n* = 11/129) areas.

The lack of financial incentive to perform vascular assessment was noted by 23 % (*n* = 86/376) of podiatrists as a significant barrier, with this generally only relevant to private practice (30 %, *n* = 87/293).

### Patient education

The majority of podiatrists (71.4 %, *n* = 269/377) reported to always provide patient education as part of a vascular assessment with very few reporting education was rarely or never provided, (3/377 [0.8 %] reported rarely providing education and 1/377 [0.3 %] reported never providing education). Main themes of patient education which emerged from open responses given included: footwear, self-care, smoking cessation, foot hygiene, exercise, daily foot inspection, first aid and signs and symptoms of PAD.

## Discussion

This is the first study to investigate the clinical indicators that podiatrists use to undertake lower limb vascular assessment and to establish the current clinical examination techniques most commonly used by podiatrists in Australia and New Zealand. We have demonstrated that pedal pulse palpation and use of Doppler were the most commonly utilised assessment methods, and that practice setting and experience had the most significant influence on performance of assessment and what type of assessment methods were utilised. This study suggests that in Australian and New Zealand podiatrists there is a reliance on subjective vascular assessment testing methods such as pedal pulses palpation and Doppler examination, and a lack of use of objective measurement such as the ABI and TBI. As objective measurements not only help to identify the presence of PAD but provide indication of severity of disease, when used in combination with signs and symptoms these tests play an essential role in guiding patient management and assessing risk status. This reliance on more subjective testing methods was more evident in private practitioners than public practitioners. This may be due to a number of different factors. The patients seen in each clinical setting tend to differ, generally with more high risk, diabetes and complex vascular pathology patients seen in public practice [17] who require more extensive investigation, which may account for some of the differences reported. In private practice, no financial incentives currently exist to complete vascular assessment and time is more limited, so practitioners may not perform the more time consuming testing such as pressure measurement.

The overall number of podiatrists reporting using the ABI on a regular basis was lower than previously reported [16] and podiatrists participating in this study reported they were more likely to use the clinical signs and symptoms of PAD present in the lower limb, as a clinical indicator to perform vascular assessment. Systemic factors, such as advanced age, smoking, cardiovascular disease and stroke, which are well-established risk factors for PAD, were much less frequently reported to be used as clinical indicators to perform such an assessment. Given that the signs and symptoms of PAD are frequently unrecognised or even absent [18], it may be likely that relying on subjective testing methods will result in missed or late diagnosis of PAD, and/or an inaccurate diagnosis of disease severity. Objective pressure measurements add another important dimension to lower limb vascular assessment, allowing for ongoing monitoring of PAD from year to year. This is particularly important for conditions such as Diabetes where changes can occur quickly and action needs to be undertaken to prevent complications such as wounds, ulceration and gangrene.

This study highlights that a large proportion of reported practices in lower limb vascular assessment being undertaken by podiatrists in Australia and New Zealand do not follow international guidelines [19] for PAD screening. However, it is likely that podiatrists are unaware of this broad guideline which recommends the use of objective pressure measurement, mainly the ABI when performing vascular assessment in populations deemed at risk of PAD. Our findings suggest the need for a podiatry specific summary of these broad international guidelines to assist podiatrists in their daily practice or increased awareness of the international guideline through continuing education.

The barriers to performing vascular assessment reported in this present study were consistent with previous studies [14, 16], with time constraints and lack of equipment most frequently cited. Uncertainty of technique was identified as a barrier to complete an assessment mainly in rural podiatrists, suggests that continuing education provision may be particularly beneficial in rural areas. A lack of equipment was identified as a major barrier in New Zealand podiatrists, however, there are differences in service provision in New Zealand compared to Australia, which may have an influence on the equipment required most frequently in daily clinical practice. Limited ability to obtain financial remuneration for vascular assessments was also a reported barrier in a quarter of all respondents. Given the importance of the task lower limb vascular assessment and it’s role in preventative care, future lobbying for health fund and/or medicare rebates may be of use to remove this barrier for podiatrists to more regularly screen for PAD in their patients who are considered at risk.

### Potential limitations

This study should be considered in light of some potential limitations. A non-validated survey was used and therefore the findings may have limited external validity and reproducibility. Despite our best efforts, our sample size was limited and may not be representative of the entire population of podiatrists in Australia and New Zealand. Over-reporting and under-reporting are possible, however piloting of the survey assisted in formulating specific answering methods and we believe this may have reduced the likelihood of this. There are also some differences in delivery of podiatric services between Australia and New Zealand which will differently influence barriers in performing testing which could be explored further in future research.

## Conclusion

Although our study only included a small proportion of practicing podiatrists in Australia and New Zealand, our findings suggest there is a lack of consistency in the profession regarding our approach to lower limb vascular assessment. Our results indicate there is greater scope for use of objective assessment techniques within the profession. Assessment methods employed by podiatrists appear to be guided by practice setting, practitioner experience and geographical location, rather than diagnostic utility of testing methods. There is a need for continuing education for podiatrists in the area of lower limb vascular assessment to increase awareness of accurate and appropriate vascular assessment requirements for populations at risk of PAD.

## Additional file

Additional file 1:
**Copy of survey given to participants.** (PDF 262 kb)
