# Supplementary material for: Vascular assessment techniques of podiatrists in Australia and New Zealand: a web-based survey
Source: J Foot Ankle Res. 2015 Dec 9;8:71. doi: 10.1186/s13047-015-0130-5 (PMC4673851; doi:10.1186/s13047-015-0130-5)
Supplement: Additional file 1: — Copy of survey given to participants. (PDF 262 kb) [file 13047_2015_130_MOESM1_ESM.pdf]

Participant information statement

You are invited to participate in the research project "Vascular assessment techniques amongst Podiatrists" which is being conducted by Dr Vivenne Chuter, and Ms Peta Craike from the Discipline of Podiatry at the University of Newcastle.

Why is the research being done?
The purpose of the research is to ascertain what current practice is amongst Podiatrists completing vascular assessments. This will help determine if a standard assessment is necessary in the future. This survey forms part of a Masters degree for Peta Craike, from the University of Newcastle. Dr Vivienne Chuter and Dr Alan Bray are involved in supervision of the project.

Who can participate in the research?
We are seeking registered Podiatrists in Australia and New Zealand who are currently practicing.

What choice do you have?
Participation in this research is entirely your choice. Only those people who give their informed consent will be included in the project. Whether or not you decide to participate, your decision will not disadvantage you.

If you do decide to participate, you may withdraw from the project at any time without giving a reason and have the option of withdrawing any data which identifies you.

What would you be asked to do?
If you agree to participate, you will be asked to complete the following survey.

How much time will it take?
Participation in this project will take approximately 10 minutes of your time.

What are the risks and benefits of participating?
There are no risks associated with participating in this research. At the completion of the survey you will be redirected to a separate page where, if you wish, you can enter your name and email address to enter the draw for one of 5 Westfield $100 gift vouchers. This information is kept separate to your survey responses to maintain anonymity.

How will your privacy be protected?
All data will be stored securely at the University of Newcastle by the Principal Researcher and only members of the research team will have access to this data. Data will be retained for at least 5 years.
Following completion of this study your name will be replaced by a code ensuring all your data is unidentifiable. Data will only be saved on electronic file in a coded form which de-identifies you. All data will be deleted/destroyed after 5 years. Electronic data will be stored on a password protected computer, paper-based records will be stored in a locked filling cabinet. Disposal of data will be performed in accordance with university policy (Research Data and Materials Management Procedure document number 000870)

How will the information collected be used?
The results of this study will disseminated via national and international conferences and for papers in scientific journals. Identifying information will not feature in the reporting of this research.

What do you need to do to participate?
Please read this Information Statement and be sure you understand its contents before you consent to participate. If there is anything you do not understand, or you have questions, contact the researcher.

Further information
Thank you in advance for your co-operation with this important effort. Your answers will make a significant contribution to understanding current Podiatry practice in Australia & New Zealand. If you would like a summary of the survey results, or if you have any questions about this research, please do not hesitate to contact me via email - Peta.Craike@newcastle.edu.au

***1. I have read the participant information statement and am eligible and willing to participate**

- ☐ Yes
- ☐ No

***2. Are you a registered Podiatrist and currently practicing Podiatry in Australia or New Zealand?**

- ☐ Yes
- ☐ No

Participant Information

***3. In the past week, the majority of your work has taken place in what kind of Podiatry setting?**

- [ ] Private practice
- [ ] Public sector
- [ ] Research/education
- [ ] Other (please specify)

***4. How many years have you been practicing as a Podiatrist?**

***5. Which of the following best describes your primary place of pratice?**

- [ ] Metropolitan
- [ ] Regional
- [ ] Rural

***6. Which state or territory does the majority of your practice take place in?**

- Queensland
- New South Wales
- Victoria
- South Australia
- Western Australia
- Tasmania
- Australian Capital Territory
- Northern Territory
- New Zealand

***7. What is the highest level of education you have completed?**

- Diploma
- Bachelor degree or graduate entry Masters degree
- Post Graduate Coursework
- Research Higher Degree

Assessment practices

***8. In your most recent day of clinical practice, how many vascular assessments did you perform?**

***9. Which of the following would prompt you to perform a vascular assessment? You may select multiple boxes**

- ☐ Burning feet
- ☐ Cold feet
- ☐ Discolouration of skin
- ☐ Ulceration
- ☐ Thickened nails
- ☐ Widespread anhidrosis
- ☐ Chillblains
- ☐ Night cramps
- ☐ Advanced age
- ☐ Diabetes
- ☐ Hypertension
- ☐ Dyslipidemia
- ☐ Smoking history
- ☐ Active smoking
- ☐ Raynaud's phenomena
- ☐ Poor healing
- ☐ New patient assessment
- ☐ Cardiovascular disease
- ☐ Cerebrovascular disease
- ☐ Other (please specify)

***10. Which of the following vascular assessment equipment do you have access to in your clinic? You may select multiple boxes**

- ☐ Stethescope
- ☐ Blood Pressure Cuff
- ☐ Toe pressure cuff
- ☐ Doppler without waveform display
- ☐ Doppler with waveform display
- ☐ Doppler with waveform display and print out
- ☐ Doppler with waveform display and software application
- ☐ Photoplethesmography probe (PPG)
- ☐ Automated toe pressure machine
- ☐ Automated ankle brachial index machine
- ☐ Other (please specify)

***11. What type of diagnostic testing do you usually use when performing a vascular assessment? You may select multiple boxes**

- ☐ Palpation of dosalis pedis pulse
- ☐ Palpation of posterior tibial pulse
- ☐ Temperature gradient
- ☐ Capillary refill time
- ☐ Doppler examination of pedal pulses
- ☐ Doppler waveform analysis
- ☐ Ankle brachial index
- ☐ Toe brachial index
- ☐ Absolute toe pressure

Other (please specify)

12. What is/are the main barriers in performing a vascular assessment in your practice?

- [ ] Time constraints
- [ ] No financial incentive
- [ ] Lack of equipment
- [ ] Lack of interest
- [ ] Unsure of techniques
- [ ] There are no barriers

*13. How much time do you estimate it takes you to complete a vascular assessment?

- ○ 5 minutes
- ○ 10 minutes
- ○ 15 minutes
- ○ 20 minutes
- ○ 30 minutes

Other (please specify)

*14. Do you book a vascular assessment as a seperate appointment, or is it performed within a routine visit?

- ○ As part of routine visit
- ○ As a seperate appointment
- ○ Other (please specify)

*15. Do you charge an additional fee for a vascular assessment?

- ○ Yes
- ○ No
- ○ Not Applicable

*16. Do you routinely provide patient education as part of your vascular assessment?

- ○ Always
- ○ Most of the time
- ○ Sometimes
- ○ Rarely
- ○ Never

*17. Which topics do you cover as part of your patient education?

Case Study

Please read the following short case study and answer the questions below.

You have just completed a diabetes foot assessment on your patient Bruce. Bruce is a 66 year old man whose medical history includes hyperlipidemia, hypertension and non insulin dependent diabetes (diagnosed 2003). He is currently taking Lipitor, Diabex and Atacand. He reports that is diabetes is well controlled. He checks his BSL's sparingly and reports his levels are always under 10mmol/L. His most recent HbA1c was 8.5%.

Your results are as follows:

Neurological Assessment: Monofilament 10/10 bilaterally. Vibration perception – absent bilaterally. Achilles reflexes – within normal limits.

Vascular Assessment: Dorsalis pedis pulses not palpable bilaterally, and tibialis posterior pulses palpable bilaterally. Doppler reveals monophasic dorsalis pedis pulses and biphasic posterior tibial pulses. Ankle brachial index is 1.4 on the right foot, and 1.2 on the left foot.

Musculoskeletal Assessment: Muscle testing within normal limits. Ankle joint range of motion reduced bilaterally. Midtarsal joint and 1st metatarso-phalangeal joint within normal limits. No deformities noted.

Footwear: Jogger. Outsole is well worn. Upper in fair condition. Fit well.

18. Based on these results, how would you classify Bruce's risk status?

- Low Risk
- Medium Risk
- High Risk

19. Is there any further vascular testing you would have performed in your clinic?

- Yes
- No

If yes, please specify

*20. Based on the results provided above, what is your management plan?
